# Neurobehavioral Consequences of Traffic-Related Air Pollution

**DOI:** 10.3389/fnins.2019.01232

**Published:** 2019-11-21

**Authors:** Ankita Salvi, Samina Salim

**Affiliations:** Department of Pharmacological and Pharmaceutical Sciences, University of Houston, Houston, TX, United States

**Keywords:** traffic pollution, mental health, anxiety, depression, cognitive decline, oxidative stress

## Abstract

Traffic-related air pollution (TRAP) is a major contributor to global air pollution. The World Health Organization (WHO) has reported that air pollution due to gasoline and diesel emissions from internal combustion engines of automobiles, trucks, locomotives, and ships leads to 800,000 premature deaths annually due to pulmonary, cardiovascular, and neurological complications. It has been observed that individuals living and working in areas of heavy vehicle traffic have high susceptibility to anxiety, depression, and cognitive deficits. Information regarding the mechanisms that potentially lead to detrimental mental health effects of TRAP is gradually increasing. Several studies have suggested that TRAP is associated with adverse effects in the central nervous system (CNS), primarily due to increase in oxidative stress and neuroinflammation. Animal studies have provided further useful insights on the deleterious effects of vehicle exhaust emissions (VEEs). The mechanistic basis for these effects is unclear, although gasoline and diesel exhaust-induced neurotoxicity seems the most plausible cause. Several important points emerge from these studies. *First*, TRAP leads to neurotoxicity. *Second*, TRAP alters neurobehavioral function. Exactly how that happens remains unclear. This review article will discuss current state of the literature on this subject and potential leads that have surfaced from the preclinical work.

## Introduction

The World Health Organization (WHO) deems air pollution as the fourth highest-ranking risk factor for global mortality (Vaughan, 2016). Air gets polluted when toxic substances such as organic hydrocarbons, particulate matter (PM), carbon monoxide (CO), nitrogen dioxide (NO_2_), carbon dioxide (CO_2_), and heavy metals released from natural and human activities gets mixed with the air (Crimmins et al., 2016; Frampton and Rich, 2016). According to reports by the United States Environmental Protection Agency (U.S. EPA), transportation contributes to more than half of CO, NO*_*x*_*, and CO_2_ emissions and release of heavy metals into the atmosphere (Xue et al., 2013; Adamiec et al., 2016; United States Environmental Protection Agency, 2018a). In this review, we have focused on the deleterious effects associated with the anthropogenic sources of air pollution, specifically TRAP.

In 2016, WHO reported that continuous exposures to air pollutants such as VEEs resulted in approximately 58% deaths from heart diseases and stroke, 18% deaths from respiratory disorders, and about 6% deaths from lung cancer (World Health Organization [WHO], 2018). Although there is considerable literature on the effect of VEE on the heart and lungs, exhaust-associated neurological effects remain unclear. Recent studies have explored the impact of VEE on brain physiology and mental health (Kim et al., 2010; Levesque et al., 2011). Various preclinical and clinical studies have demonstrated the effect of VEE on neurobehavioral alterations and cognitive deficits (Lim et al., 2012; Salvi et al., 2017). Although the exact physiological mechanism contributing to these outcomes is not clear, VEE-associated increase in oxidative stress and neuroinflammation and the consequent compromise in normal neuronal circuit functions are postulated to be the potential contributors to abnormal behavioral and cognitive output (Hovatta et al., 2010; Grases et al., 2014). Oxidative stress is specially considered to be one of the primary factors mediating VEE-associated health effects because the primary constituents of VEE such as CO_2_, CO, and NO_2_ are pro-oxidant in nature (Hartz et al., 2008; Lodovici and Bigagli, 2011). Present discussion focuses on the neurobehavioral consequences of TRAP and the involvement of oxidative stress in mediating these effects.

## Vehicle Exhaust Emissions

Exhaust emissions are released from vehicles during their operation, refueling, manufacturing, and disposal (International Agency for Research on Cancer, 2014). These emissions are primarily of two kinds, gasoline and diesel emissions, both of which are composed of CO_2_, CO, NO_2_, hydrocarbons, heavy metals, and PM (Pulles et al., 2012; Xue et al., 2013). According to reports by the U.S. EPA, approximately 4.6 metric tons of CO_2_ is emitted by a passenger vehicle every year (United States Environmental Protection Agency, 2018b). Another report suggests that 75% of CO pollution in the United States comes from vehicles (Brinson, 2019). In megacities, vehicles contribute to roughly 16–39% of ambient PM_2_._5_ levels (Ito et al., 2004). Reports indicate that levels of heavy metals such as lead, copper, and zinc on highways significantly exceed their maximum concentrations in soil, indicating the growing intensity of heavy metal pollution from transportation (Sezgin et al., 2004). Individuals living or working in close proximity to freeways are exposed to these exhaust constituents on a prolonged basis. Occupational exposure to VEE is observed in traffic policemen, ground facility workers at airports, and professions involving vehicle maintenance and heavy equipment operation (Hoek et al., 2002; Janssen et al., 2003). Continuous exposures to constituents of VEE are associated with several deleterious effects (Calderon-Garciduenas et al., 2008b; Huls et al., 2019). Cardiovascular (Minami et al., 1999; Brunekreef and Holgate, 2002), respiratory (Brightwell et al., 1989; Holgate et al., 2003), and carcinogenic (Mohr et al., 1976; Muzyka et al., 1998) effects associated with VEE have been investigated for quite some time, however, attention to exhaust-associated neurological effects is fairly recent.

Several studies link VEE to pathology of dementia-related disorders such as Alzheimer’s disease and Parkinson’s disease (Calderon-Garciduenas et al., 2004; Levesque et al., 2011; Heusinkveld et al., 2016). Prenatal DEE was reported to cause alteration in dopamine levels in the prefrontal cortex (PFC) of exposed children, resulting in motor impairments (Yokota et al., 2009; Suzuki et al., 2010). Another epidemiological study suggested that elderly population with preexisting cardiovascular conditions exhibit increased susceptibility to exposure-associated stroke (Yorifuji et al., 2013). Although most of these studies focused on VEE-associated stroke (Block and Calderon-Garciduenas, 2009; Vidale et al., 2010) and dementias (Calderon-Garciduenas et al., 2008a; Wang et al., 2009), the past few years have shed more light on the effect of prolonged VEE on an individual’s psychology, mood, and behavior (Salvi et al., 2017; Newbury et al., 2019).

## Psychological Impact of Vehicle Exhaust Emissions

Reports by the National Institute of Mental Health suggest that one in five individuals suffer from a mental illness every year (National Institute of Mental Health, 2019). Approximately 31.1% adults in the United States have anxiety disorders (National Comorbidity Survey, 2017a, b). The estimate for individuals suffering from depression is about 16 million/year (2019). Mental illnesses have led to a large number of hospitalizations, high rates of suicides, and an overall economic burden on our society (Walker et al., 2015). Therefore, preserving mental health has become one of the primary objectives of the health-care industry.

Environment is purported to play a major role in the etiology of various mental disorders. Environmental factors at birth, such as winter and spring births, maternal psychological stress during pregnancy increase the risk of getting schizophrenia in genetically susceptible individuals (Schmidt, 2007). Similarly, exposure to environmental toxicants, such as dioxin, and secondhand tobacco exposure are associated with behavioral alterations such as increased aggressiveness (Evans, 2003; Chin, 2010). Therefore, it is reasonable to hypothesize that continuous exposure to VEE can potentially adversely affect the mental state of an individual.

### Clinical Studies

Recent surveys suggest that individuals living or working near freeways and heavy traffic areas who get exposed to VEE on a daily basis show a higher risk of developing mental comorbidities such as depression and cognitive decline (Adler, 2013). In 2015, WHO reported an association between VEE exposure and heightened anxiety symptoms in over 70,000 women (Power et al., 2015). In a study conducted on geriatric population living in Korea with high ambient levels of PM, NO_2_, and ozone, increased depressive symptoms were seen in the elderly population as compared with those living in areas with a lower level of pollutants (Lim et al., 2012). Similarly, a study in Mexico City suggested that children living in the polluted urban areas of the city performed poorly in cognition tasks despite having no genetic or hereditary susceptibility to mental disorders. Brain magnetic resonance imaging (MRI) studies on these children depicted lesions in their PFC, potentially responsible for the observed cognitive dysfunction (Calderon-Garciduenas et al., 2008a). Prolonged exposure to heavy metals from TRAP such as lead, cadmium, and zinc have been associated with increased risk of Parkinson’s disease, Alzheimer’s disease, and autism spectrum disorders (ASDs) (Palacios et al., 2014; Bjorklund et al., 2018; Qin et al., 2018).

Traffic-related air pollution exposure during pregnancy and early life stages is reported to have a significant impact on brain development. In 2016, researchers at the Columbia Center for Children’s Environmental Health reported that prenatal exposures to VEE could influence the mental development in children resulting in poor social skills and difficulty managing emotions (Perera, 2016). In fact, several epidemiological studies report that prenatal and neonatal exposures to TRAP lead to delayed cognitive development and increased risk of ASD and schizophrenia (Windham et al., 2006; Oudin et al., 2016). A clinical study conducted on 2,063 individuals in the age group of 16–18 years indicated that developmental TRAP exposures are often associated with adolescent psychotic experiences (Newbury et al., 2019). Another study provided support for association between early life exposure to TRAP and cognitive decline in older adults (Tonne et al., 2014). This suggests that TRAP exposure during early stages of development is associated with mood and cognitive disorders that persist from childhood through late adulthood.

### Preclinical Work

At the preclinical level, there is paucity of information on the direct effect of VEE on psychological disorders. Challenges involved in mimicking an environment similar to human TRAP exposure without stressing the animals and then evaluating behavioral patterns are the potential reasons for a smaller number of studies. For instance, motors used to generate VEE are loud and are likely to induce stress in animals, thereby affecting their performance in behavioral tests (Castelhano-Carlos and Baumans, 2009). Moreover, behavioral tests used to assess anxiety-like and depression-like behaviors as well as those assessing cognitive performance are sensitive measures and only act as indicators of mood and cognition in animal models. Their accuracy often depends on factors such as noise in test facility, baseline stress levels, and preexisting health conditions of subject animals. For instance, forced swim test used to measure depression-like behaviors relies on the ability of test animals to swim and would be biased if the animal suffers from motor impairments. These considerations make it difficult to evaluate VEE-associated behavioral alterations.

Within the existing literature, one study indicated that prenatal exposure to DEPs via subcutaneous injections of DEP suspensions leads to serotonergic neuronal activation of the dorsal raphe nucleus eventually resulting in an increased anxiety-like behavior in male offspring (Yokota et al., 2016). In a SVEE model in rats, whole-body exposures to gaseous constituents of exhaust, namely, CO_2_, CO, and NO_2_, resulted in elevated anxiety-like and depression-like behaviors and memory deficits in exposed rats (Salvi et al., 2017). Another study in mice depicted that the combined effect of PM and dim light at night leads to increased anxiety-like and depression-like behaviors in exposed mice (Hogan et al., 2015). Similarly, whole-body exposure to nanoscale PM derived from DEP in adult mice led to increased anxiety-like and depression-like behaviors (Ehsanifar et al., 2019). Postnatal exposure to ultrafine PM has been reported to result in an altered social behavior (Sobolewski et al., 2018) as well as motor and memory function in mice (Cory-Slechta et al., 2018). Prolonged exposures to high levels of lead in pregnant rats led to neurotoxicity in fetuses, indicated by Purkinje cell degradation and lack of cerebellar development (Saleh et al., 2018).

There are a greater number of studies that report the negative effect of VEE on cognition in animal models. For instance, a study in mice reported that whole-body exposure to nanoparticle-rich diesel exhaust impairs spatial learning and memory function in exposed mice (Win-Shwe et al., 2012). Another study reported decreased learning-memory function in male mice in the Moris water maze test following prenatal DEE via subcutaneous injection. Interestingly, the same study did not show any difference in learning function of exposed mice in passive avoidance test (Yokota et al., 2015). Such variability in tests is often attributed to the order in which the tests were conducted. Another confounding variable in preclinical studies investigating the effect of VEE is the medium of exposure. Administering exhaust particles in the form of subcutaneous injections (Yokota et al., 2016) or via inhalation exposure (van Berlo et al., 2010) subjects the rodents to an extra level of stress and could affect the results of behavioral tests. On the contrary, VEE exposures in whole-body exposure chambers potentially offer a relatively controlled medium of exposure that does not subject the animals to secondary stress during exposure.

Sex differences are another important variable. Prenatal whole-body exposures to nanosacale PM (<100 nm) led to decreased immobility in tail suspension test, indicative of a depression-like behavior in male offspring, but not in female offspring. The test depicted higher baseline immobility in females, which could partially explain why no significant effect was seen in exposed female mice (Davis et al., 2013). This observation agrees with previous reports that support a higher prevalence of a depression-like behavior and altered social interaction in females (Parker and Brotchie, 2010; Liu et al., 2019). More studies are needed to understand the mechanical basis for these sex-dependent differences in VEE-associated impairments.

Despite variability and limitations, preclinical studies are significant, as they present important information with regard to the possibility of occurrence of VEE-associated neurobehavioral deficits.

## Physiological Mechanisms

To understand the underlying pathological mechanisms potentially responsible for VEE-associated neurobehavioral alterations, various brain regions were studied extensively. It was suggested that pollutants could affect the brain via (a) olfactory tract, (b) signal transmission between the brain and gastro-intestinal tract (brain–gut axis), and (c) blood–brain barrier (BBB) (Block et al., 2012). Therefore, investigating the effect of VEE on these regions is crucial in understanding exhaust-related effects on brain. For instance, it was reported that pollutants disrupt the structure and integrity of the BBB. It was observed that sub-chronic exposure of mice to VEE led to increase in BBB permeability and oxidative stress in the cerebral vessels of the mice (Oppenheim et al., 2013). In fact, alteration in BBB permeability is often associated with ischemic stroke (Kuroiwa et al., 1988; Latour et al., 2004) and could partly explain TRAP-associated stroke incidences (Lisabeth et al., 2008; Vidale et al., 2010).

Activation of neuroinflammatory pathways following exposure to VEE is another well-investigated area (Hougaard et al., 2008; Ehsanifar et al., 2019). In a whole-body exposure study in mice, DEP resulted in activation of neuroinflammatory markers such as tumor necrosis factor-α (TNF-α) and interleukins (ILs) (IL-6 and IL-1β) in the midbrain. In the same study, neurodegenerative markers associated with pathology of Alzheimer’s disease such as amyloid beta-42 and tau proteins were found to be elevated in the frontal and temporal lobes, whereas α-synuclein, the hallmark protein found in Lewy bodies associated with Parkinson’s disease, was found to be elevated in the midbrain (Levesque et al., 2011). Both the midbrain and temporal lobe have been implicated in schizophrenia (Nopoulos et al., 2001; Howes et al., 2013), depression (Paradiso et al., 2001; Friedman et al., 2014), Alzheimer’s disease (Lee et al., 2015), and Parkinson’s disease (Martin et al., 2009). The enzymes involved in mediating oxidative stress mechanisms have been found to be elevated in the frontal cortex and the hippocampus with high levels of TRAP (de Lima et al., 2005; Peters et al., 2006). In fact, it is theorized that oxidative stress may be a contributing factor for VEE-associated morbidity.

## Oxidative Stress and Vehicle Exhaust Emissions

*Oxidative stress* results from an imbalance between the production of reactive oxygen species (ROS) and reactive nitrogen species (RNS) and endogenous antioxidant defense systems (Betteridge, 2000). Oxidative stress is considered to be one of the primary underlying mechanisms that are linked to a myriad of diseases such as diabetes, liver diseases, atherosclerosis, Parkinson’s and Alzheimer’s disease, schizophrenia, and pulmonary fibrosis (Cheresh et al., 2013; Li et al., 2014; Giulia et al., 2017).

Interestingly, there are several studies that have suggested a causal link between oxidative stress in the PFC, hippocampus, and amygdala regions of the brain and neurobehavioral impairments (Patki et al., 2013; Grases et al., 2014; Solanki et al., 2017). Studies indicate that anxiety and depressive disorders are associated with reduced levels of key antioxidants such as glyoxalase-1 and glutathione-*S*-reductase-1 (Gałecki, 2014; Hassan et al., 2014).

There are different circuits in the brain that regulate mood and cognition. For instance, synaptic transmission through the limbic-cortical-striatal-pallidal-thalamic (LCSPT) circuits, which comprise the PFC, amygdala, hippocampal subiculum, ventromedial striatum, mediodorsal and midline thalamic nuclei, and ventral pallidum, regulate emotion (Ongur et al., 2003). Lesions in the regions that make up the LCSPT circuit disrupt transmission resulting in symptoms observed in depressive and mood disorders (Drevets, 2004). Similarly, the medial prefrontal network regulates attention and other emotional stimuli associated with anxiety disorders (Gusnard et al., 2001). Disruption of synaptic transmission through circuits involving hippocampus such as perforant pathway and Schaffer collateral pathway has been associated with learning and memory disorders (Akil and Lewis, 1993). ROS- and RNS-induced alterations in the above-mentioned regions and circuits have been implicated in various mental disorders (Hovatta et al., 2005; Andreazza et al., 2013).

Vehicle exhaust emission is considered to be one of the critical sources of oxidative stress (Hartz et al., 2008; Lodovici and Bigagli, 2011). Among the constituents of VEE, the three primary gaseous constituents, namely, CO_2_, CO, and NO_2_, are pro-oxidant in nature (Westerholm and Egeback, 1994). Pro-oxidants are agents that generate ROS and RNS via oxidation. In 2015, the U.S. EPA published a study where they observed that short-term exposures to VEE lead to an increased level of oxidative stress in asthmatic and healthy adults. It was observed that DEE leads to damage of human microvascular endothelial cells and an increased level of ROS in the cells (Tobwala et al., 2013). DEP exposures in mice have also been associated with activation of ROS and elevation of mRNA expression of pro-inflammatory cytokines IL-6 and IL-β in microglia (Roque et al., 2016).

Among the pro-oxidant constituents of VEE, CO_2_ induces hypoxic condition in the body, which results in oxidative stress (Bentes de Souza et al., 2004). CO forms complexes with reduced transition metals such as Fe^2+^, eventually forming ROS and RNS (Piantadosi, 2008). Furthermore, CO inhibits cytochrome bc1 complex of the mitochondrial electron transport chain (ETC) and results in superoxide generation, thus contributing to oxidative stress (Zhang and Piantadosi, 1992). NO_2_ oxidizes into NO_2_ radical upon inhalation, which then not only causes lipid and protein oxidation but also inhibits endogenous antioxidants, thus further aggravating oxidative stress (O’Neill et al., 1995). Therefore, considering the correlation between VEE, oxidative stress, and the brain, the effect of pro-oxidant constituents of vehicle exhaust on behavior and cognition must be investigated.

## Conclusion

In summary, air pollution is a serious environmental hazard and could contribute to psychiatric ailments including decreased cognitive functions, depressive symptoms, and neurodegenerative pathologies. Human epidemiological studies indicate that individuals living and working in areas of heavy vehicle traffic are more susceptible to these conditions. Although pathological alterations leading to these conditions are unclear, animal studies have provided useful insights into the mechanistic basis behind TRAP-mediated health effects, especially pollution-related neurotoxicity that leads to increase in oxidative stress and neuroinflammation. On the other hand, lack of reliable simulation models and confounding variables, including medium of exposure, sensitivity of behavior tests, and sex differences, act as limiting factors to conduct an objective assessment of TRAP-associated deleterious effects. Despite this, current literature lays a solid groundwork that can be used to develop more studies that will help further understand the effect of TRAP on the brain.

## Author Contributions

AS conducted literature review and wrote the first draft of the manuscript. SS finalized the draft after several layers of edits and iterations.

## Conflict of Interest

The authors declare that the research was conducted in the absence of any commercial or financial relationships that could be construed as a potential conflict of interest.

## Abbreviations

DEE: diesel exhaust exposure
DEPs: diesel exhaust particles
SVEE: simulated vehicle exhaust exposure
TRAP: traffic-related air pollution
VEEs: vehicle exhaust emissions.
